# Risk factors for severe hypocalcemia after parathyroidectomy in dialysis patients with secondary hyperparathyroidism

**DOI:** 10.1038/s41598-018-26142-9

**Published:** 2018-05-17

**Authors:** Xiaoliang Sun, Xiaoqing Zhang, Yao Lu, Ling Zhang, Meng Yang

**Affiliations:** 1 Department of General Surgery, China-Japan Friendship Hospital, 100029 Beijing, China; 2 Department of anesthesiology, Peking University Third Hospital, 100191 Beijing, China; 3 Department of Nephrology, China-Japan Friendship Hospital, 100029 Beijing, China

## Abstract

Severe hypocalcemia (SH) is a common and serious complication in dialysis patients with secondary hyperparathyroidism (SHPT) after parathyroidectomy (PTX). The aim is to explore the risk predictors of SH in post-PTX dialysis ESRD patients with SHPT. 129 consecutive dialysis patients with SHPT underwent PTX were retrospectively reviewed. A total of 22 clinical parameters were included in the study. SH was defined as the minimum values of serum calcium lower than 1.875 mmol/L (7.5 mg/dL) after surgery. Univariate analysis showed that pruritus, lumbar X-ray changes of renal osteodystrophy, pre- and post-operative intact parathyroid hormone (iPTH), Calcium, alkaline phosphatase, and gland mass were significantly different between SH and non-SH groups. In the multivariate logistic regression model, the pre-operative serum iPTH, calcium, and pruritus were independent risk predictors of SH. AUCs for pre-operative serum iPTH, calcium and pruritus were 0.810, 0.714 and 0.591, respectively. Patients with higher level of pre-operative serum iPTH, lower level of serum calcium and with no/mild symptoms of pruritus are at greater risk of developing SH after PTX.

## Introduction

Secondary hyperparathyroidism (SHPT) is a complication of chronic kidney disease (CKD), especially common in patients with end-stage renal disease (ESRD)^1^. Severe SHPT causes bone pain, muscle weakness, and itching. Its association with disturbances in mineral and bone metabolism are one of the most prominent risk factors for death and cardiovascular events. For patients with ESRD who refractory to aggressive medical therapy, parathyroidectomy (PTX) is the widely-acknowledged method in treating those patients with severe SHPT. However, post-operative hypocalcaemia is the most common medical complication following PTX^1,2^.

Literature reported the incidence of post-PTX hypocalcemia in SHPT patients ranges between 72–97% despite frequent monitoring of the serum calcium level and adjustments of the calcium and vitamin D supplements^3^. Severe hypocalcemia (SH) can lead to tetany, seizure, cardiac arrhythmia and even sudden death.

Therefore, early identification of risk factors and preventative calcium supplement can help pre-empt hypocalcemia and avoid serious consequences. However, relevant studies addressing this issue are scanty and heterogeneity in nature, and results are conflicting. Most studies concentrated only on a few clinical parameters that could not reflect the comprehensive characteristics of the related risk factors.

The aim of the current study is to help explore the relationship between possible comprehensive relevant clinical parameters and the occurrence of SH in post-PTX dialysis ESRD patients with SHPT. And these screened risk factors could be utilized in intensive surveillance and preventative guidance in calcium medical treatment in the early postoperative period.

## Methods

### Patients data

We retrospectively reviewed a total of 129 SHPT patients with ESRD needing regular renal dialysis who had received PTX in China-Japan Friendship Hospital from February 2017 to November 2017. And we performed the most comprehensive data collection. Baseline data included age, gender, height, body weight, BMI, and the clinical data of presenting symptoms (pruritus, insomnia, height shortening, ostealgia, skeleton deformity, fracture), vintage of dialysis, dialysis modality, lumbar X-ray of renal osteodystrophy (described as “vertebral hyperosteogeny, fuzzy edge of the vertebral body, narrowing of intervertebral space, etc.” with symptoms of osteoporosis, skeleton deformity or height shortening, and diagnosed by two independent experienced radiologists). Laboratory data of serum concentration of pre- and post-operative intact parathyroid hormone (iPTH and post-iPTH), pre- and post-operative Calcium (Ca and post-Ca), pre-operative phosphorus (P), Ca × P and alkaline phosphatase (ALP) were recorded.

For prutitus symptom, Patients were asked to indicate the extent to which they were bothered by itchy skin as: not at all or somewhat bothered, moderately or extremely bothered. Somewhat bothered was intermediate between not at all bothered and moderately bothered, therefore, it was defined as mild itchiness for the analyses^4^. Patients were grouped according to not at all or somewhat bothered (No/Mild) or moderately or extremely bothered (Severe).

Persistent HPT was identified if the lowest post-operative iPTH concentration was still higher than the upper normal limit measured after initial PTX on post-operative day one. In the current study, parameter of post-iPTH was noted as categorical variable of less than 88 pg/ml (upper normal range)^5^. As the predictive value of total volume and mass of dissected parathyroid gland (PTG) were inconclusive, we recorded these intra-operative data for further analysis. In order to generalized the data, all patients were enrolled regardless of the success of the PTX surgery. This study was approved by the ethics review board of China-Japan Friendship Hospital and was performed in accordance with relevant guidelines and regulations. And the patients’ informed consents have been obtained.

### Data availability

The datasets generated during and analyzed during the current study are available from the corresponding author on reasonable request.

### Surgical indications

The surgical indications in our hospital for the ESRD patients with SHPT who are: (1) persistent elevation of iPTH of greater than 800 pg/ ml; (2) uncontrolled hypercalcemia with hyperphosphatemia or clinical symptoms of SHPT refractory to medical treatment; (3) resistance to active vitamin D in the past medical history; (4) power-Doppler images provide the evidence of more than one PTG hypertrophy with diameter ≥ 1 cm, and rich in blood flow.

### Parathyroid gland management

All operations were performed by expertise PTX surgeons. All resected PTG were measured, weighted and verified histologically. The volume of each PTG was estimated by using the formula: a*b*c*π/6 mm^3^ (where a, b, c are the dimensions of gland in millimeters)^6^. The final diagnosis of the pathological report was used to correct the actual number, and accordingly volume and mass of identified PTG.

### Definition of hypocalcemia

SH after surgery was defined as a minimum value of serum calcium level lower than 1.875 mmol/L (Ca < 7.5 mg/dL) postoperatively within 2 days as symptoms of hypocalcemia develop^7^. In clinical practice, serum calcium concentration lower than this level is a strong indicator for initiation of calcium treatment. In our center, intravenous infusion of calcium was initiated when patients with serum calcium lower than 1.8 mmol/L or with symptoms of hypocalcemia; Otherwise, other patients with low calcium level were treated with oral intake of calcium supplements. The doses of medications were adjusted according to the clinical symptoms and laboratory data. Therefore, in the current study, SH and non-SH were grouped by calcium concentration first day after surgery in the morning.

### Statistics

The statistical analysis was performed using SPSS 22.0 version (SPSS Inc., Chicago, IL, USA). Continuous data with normal distribution were presented as means ± SD and categorical variables, as numbers or percentages. Student’s *t* test was used to compare continuous variables and Chi-square test for categorical variables. The non-normally distributed continuous variables were presented as median and interquartile range (IQR) and compared using the Mann-Whitney *U* test. Variables included age, gender, height, body weight, BMI, vintage of dialysis, dialysis modality, presenting symptoms (pruritus, insomnia, height shortening, ostealgia, skeleton deformity, bone fracture), lumbar X-ray of renal osteodystrophy; laboratory variables included iPTH, post-iPTH, Ca, post-Ca P, Ca × P and ALP; Intraoperative variables included total mass and weight of dissected PTG. Covariates in the univariate analysis that reached statistical significance were chosen for further multivariate logistic regression analysis model. A *P* value  < 0.05 was considered significant. Receiver operating characteristic (ROC) curve analysis was further performed to depict the Area under the ROC curve (AUC) in order to help better evaluate the diagnostic value of the selected variates.

## Results

**Figure 1:**
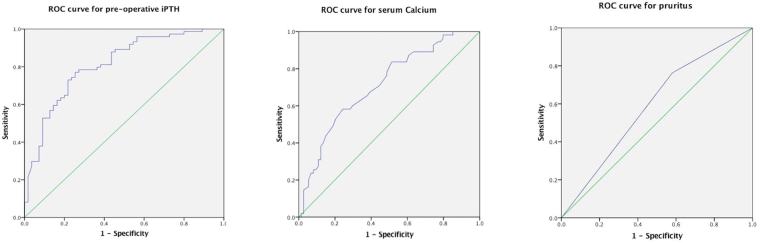
ROC curves for serum iPHT, serum calcium and symptom of pruritus. This graph shows the ROC curves for pre-operative iPTH (**a**), serum calcium level (**b**) and symptom of pruritus (**c**). AUC for pre-operative serum iPTH level was 0.810 (95% CI = 0.735 to 0.885); AUC for serum calcium level was 0.714 (95% CI = 0.625 to 0.802); AUC for pruritus was 0.591 (95% CI = 0.493 to 0.690). ROC, receiver operating characteristic; AUC, area under the ROC curve; CI, confidence interval.

The baseline data of patient characteristics, presenting symptoms, preoperative laboratory and imaging parameters and operative findings are shown in Table 1. A total of 74 patients (57.4%) develop SH after PTX, with variates of pruritus (P = 0.031), lumbar X-ray changes of renal osteodystrophy (P = 0.025), serum iPTH (P = 0.000), post-iPTH (P = 0.016), Ca (P = 0.000), ALP (P = 0.000), and PTG mass (P = 0.046) were significantly different between SH and non-SH groups. In the multivariate logistic regression model, the preoperative serum Ca, iPTH, and pruritus were independent predictors of SH postoperatively. Patients with higher level of pre-operative serum iPTH, lower level of serum calcium and without symptoms of pruritus are at greater risk of developing SH after PTX. (Table 2). ROC curves were performed for pre-operative serum iPTH, calcium and pruritus. The AUCs for these two prognostic risk factors were 0.810 (95% CI = 0.735 to 0.885), 0.714 (95% CI = 0.625 to 0.802) and 0.591 (95% CI = 0.493 to 0.690), respectively (Fig. 1). And as independent risk factors, pre-operative serum iPTH and serum calcium are more prominent than symptom of pruritus.

**Table 1:** Comparison of relevant clinical parameters between patients with and without postoperative hypocalcemia (Ca < 1.875 mmol/L)

	All patients	Patients with post-operative Severe Hypocalcemia		*P* value
	N = 129	Yes (SH group)	No (non-SH group)	
		N = 74	N = 55	
Female	69	40	29	0.881
Age (years)	48 (40, 55)	47(37, 54)	49 (43, 57)	0.508
Vintage of dialysis (months)	97.36 (72,120)	94.13(72,120)	101.64(72,120)	0.292
HD modality (HD/PD)	121/8	71/3	50/5	0.241
Height (cm)	164 ± 8	163 ± 8	165 ± 8	0.167
Weight (kg)	59.1 ± 12.2	57.4 ± 10.9	61.4 ± 13.5	0.065
BMI	21.99 (19.1,24.15)	21.59(19.1,24.13)	22.53(19.5,25.3)	0.328
**Presenting symptoms**				
Bone pain (n,)	115	66	49	0.986
Severe Pruritus (n)	85	43	42	0.031*
Insomnia (n)	77	43	34	0.671
Height shorting (n)	55	37	18	0.05
Bone deformity (n)	45	29	16	0.234
Fracture (n)	12	10	2	0.056
Lumbar X-ray of renal osteodystrophy (n)	71	47	24	0.025*
**Laboratory examination**				
Pre-operative iPTH (pg/ml)	2041.13 (1187,3214.65)	2502.99 (1721.68,3383)	1419.73 (815,1730.4)	0.000*
Post-operative iPTH (<88 pg/ml) (n)	106	66	40	0.016*
Serum Calcium (mmol/L)	2.49 ± 0.20	2.43 ± 0.20	2.57 ± 0.18	0.000*
Serum phosphorus (mmol/L)	2.16 ± 0.56	2.15 ± 0.57	2.18 ± 0.56	0.798
Calcium-Phosphorus Product	5.34 ± 1.52	5.17 ± 1.57	5.57 ± 1.44	0.139
ALP (U/L)	625.09 (166.5,899)	881.16 (434.25,1300.25)	280.55 (112,246)	0.000*
**Parathyroid glands**				
Volume of excised glands (cm^3^)	2.35 (1.30,2.81)	2.59 (1.51,3.07)	2.02 (1.08,2.52)	0.05
Mass of excised glands (g)	2.89 (1.56,3.4)	3.06 (1.88,3.62)	2.46 (1.32,3.09)	0.046*

HD, hemodialysis; PD, peritoneal dialysis; iPTH, intact parathyroid hormone; ALP, alkaline phosphatase; BMI, body mass index; SH, post-operative patients with severe hypocalcemia; non-SH, post-operative patients without severe hypocalcemia.

^*^P value < 0.05.

Continuous data with normal distribution were presented as Mean ± SD and categorical variables, as numbers or percentages.

Student’s *t* test was used to compare continuous variables and Chi-square test for categorical variables.

The non-normally distributed continuous variables were presented as median and interquartile range (IQR) and compared using the Mann-Whitney *U* test.

**Table 2:** Multivariate logistic regression analysis for the development of severe hypocalcemia (Ca < 1.875 mmol/L) after parathyroidectomy.

Variables	Wald	OR	*P* value
Serum Calcium (mmol/L)	7.942	44.492	0.005*
Pre-operative iPTH (pg/ml)	4.047	0.044	0.044*
Severe Pruritus (n)	3.944	0.334	0.047*
ALP	3.285	0.07	0.070
Mass of excised glands (g)	1.425	0.233	0.233
Post-operative iPTH (<88 pg/ml) (n)	1.002	0.591	0.317
Lumbar X-ray of renal osteodystrophy	0.003	0.957	0.957

iPTH, intact parathyroid hormone; ALP, alkaline phosphatase.

^*^P value < 0.05 was considered statistically significant.

Covariates in the univariate analysis that reached statistical significance were chosen for multivariate logistic regression analysis model.

The selected variables include: pruritus, lumbar X-ray of renal osteodystrophy, pre-operative iPTH, post-operative iPTH, serum Calcium, ALP, and mass of excised glands.

## Discussion

The prevalence rate of SHPT (iPTH > 1000 pg/ml) in dialysis patients was 10.7% according to Brazilian Census of Parathyroidectmy^8^. A statistical survey of dialysis conducted by the Japanese Society for Dialysis Therapy reported that approximately 10% of patients received PTX after 10 years of dialysis, and the ratio reached to 30% when the dialysis period lasted for more than 20 years^9^. In China, a cross-sectional surgery showed the prevalence of chronic kidney disease was 10.8%^10^. Data from Dialysis and transplantation registration system revealed that about 200 million were on dialysis in China, and the rate of SHPT patients is still rising^11^.

PTX is regarded as an ultimate therapeutic resource in severe SHPT patients. In recent years, patient with severe SHPT can benefit from surgical approach. PTX offers a long-term survival advantage over medical therapy for severe SHPT^12^. However, SH remains a frequently encountered post-PTX complication and its prevention represents a challenge as severe SH could cause lethal consequences. It was postulated that multiple risk factors were related with post-PTX SH. However, little relevant studies have explored its relationship with prognostic risk factors and these studies were heterogeneity in nature with conflicting results.

Since our hospital is one of the biggest and most comprehensive therapeutic center for ESRD patients in China, we have multidisciplinary team including nephrology department, cardiology department, intensive care unit, anesthesia department and general surgery department to make the most effective therapeutic regime. In the current study, we performed a retrospective study with all potential predictors in our center to explore the potential clinical parameters associated with the occurrence of SH after PTX in ESRD patients needing regular dialysis with refractory SHPT. By using multivariate logistic regression analysis, we identified the preoperative serum Ca, iPTH, and pruritus as independent risk predictors for the occurrence of SH following PTX.

The results of lower preoperative serum Ca and higher iPTH level as independent risk factors were in accordance with several other studies as the SH after PTX could be partially explained by a higher base-line bone-remodeling status, a rapid shift of calcium from the circulation to the skeletal system due to hypothyroidism after PTX, and extensive remineralization of the skeleton^1,3,13^. Pre-operative PTH was a significant indicator for post-PTX SH. Under stimulation with excess iPTH, bone formation and bone resorption are both increased despite a marked negative balance. iPTH increases bone resorption and decreases bone formation; therefore, the reversed effect would happen shortly after PTX, and hypocalcemia would develop in accordance with this change immediately after PTX^14^. In China, most SHPT patients needing PTX were at the end-stage of the disease and with severe symptoms and delayed surgical opportunity. In this study, the pre-operative iPTH of these patients were significantly higher than the values of SHPT patients in developed countries. Further studies should concentrate more on the demographic characteristics of this specific population.

A novel correlation between pruritus and the SH post-PTX was identified in our study. A total of 85 patients developed symptom of pruritus with 43 in the SH group. In the multivariate logistic regression analysis, the pruritus was an independent predictor for the development of SH post-PTX. Uremic pruritus, which is a common symptom for patients with kidney failure, affecting up to 46% of hemodialysis patients. It has been identified as a key research priority by patients with kidney disease^15^. The pathophysiology is not fully understood and likely to be multifactorial. Many metabolic factors have been implicated in the pathogenesis of itching, for example, hypercalcemia, hyperphosphatemia, secondary hyperparathyroidism, and hypermagnesemia^16^. A multivariate logistic model was used to determine the relationship between patient characteristics and laboratory values with the likelihood of developing moderate/extreme pruritus *vs.* mild/no pruritus in the combined Dialysis Outcomes and Practice Patterns Study sample showed: higher serum calcium or phosphorus levels, and calcium phosphorus product concentrations had significantly higher odds of having moderate/extreme pruritus^4^. In SH post-PTX group, more patients developed pruritus with higher calcium level before operation. Variables of serum Ca and pruritus skin were all independent risk predictors for SH in multivariate logistic regression analysis.

The differences of mass of PTG, pre-operative ALP level, and lumbar X-ray changes between SH and non-SH groups were significant with univariate analysis. While these three factors were not independent risk predictors of SH post-PTX according to multivariate logistic regression analysis model.

As iPTH levels might be induced by the pharmaceutical drug under manageable condition, whereas the volume and weight of PTG is to a large extent of more importance on the regulation of iPTH secretion and appropriate guidance. Li Fang suggested that the larger PTG might secret more iPTH, confirming that the volume and weight of PTG could be used as an indication for surgical treatment^6^. However, contradictory finding was reported as total mass and blood supply of PTG and the secretion of iPTH were not correlated^6,17^. In our study with univariate analysis, the mass and volume of PTG was not recognized as independent predictors for SH post-PTX in the multivariate regression analysis model. Further studies are warranted in demonstrating the predictive role of the related parameters.

Pre-operative ALP level was reported as an independent risk factor for prediction of SH in some literature^1,3^. It was postulated that the serum ALP level might serve as a biomarker indicating the intensity of bone formation and the likely calcium requirement of individual patients. However, the results remained controversial concerning different studies. In a case series, the high preoperative ALP was not shown to be predictive of hypocalcemic as being presented by a prolonged hospital stay^18^. In the current study, the univariate analysis of pre-operative ALP level of the SH group were significantly different. However, in the multivariate logistic regression analysis, its contribution was not as significant as other parameters such as serum Ca, iPTH and pruritus. This might be explained by the intricate cross-impact of the other analyzed multivariate or the existence of confounder, and the serum ALP level was not an independent risk factor itself in predicting the appearance of SH after PTX.

47 patients in SH group and 24 patients in non-SH group developed renal osteodystrophy demonstrated in lumbar X-ray. Chronic kidney disease or renal injury impairs skeletal anabolism, thereby decreasing osteoblast function and bone formation rates. Loss of osteoblastic bone formation due to renal injury shrinks the size of the rapidly exchangeable phosphate and calcium pools causing early stimuli for SHPT. SHPT on skeletal remodeling may represent adaptive changes following renal injury. In ESRD, the sustained increase in iPTH levels results in an unwanted disorder of skeletal remodeling, a high turnover renal osteodystrophy. If hyperparathyroidism is prevented or treated, a low turnover osteodystrophy, the adynamic bone disorder, is observed^19,20^. The pathophysiological mechanism underlying the post-PTX hypocalcemia is and increased shift of calcium from the circulation to the bone tissues^13^.

For patients with renal osteodystrophy, concentration of iPTH would fall dramatically after PTX. The concentration of serum calcium which was influence by the iPTH level, would decrease correspondingly. In some cases, SH may ensue.

The strength and originality of the current study includes: first, the sample size is large in comparison to other similar studies; second, we analyzed the most comprehensive relevant factors and using multi-variate regression analysis to select the most prominent independent risk factors; third, this study for the first time evaluate the correlation between post-operative hypocalcemia and clinical symptoms, mass and weight of dissected thyroid tissue, body mass index, and lumbar radiological findings. Finally, we performed the ROC curves and calculate the AUCs for pre-operative serum iPTH and calcium level in order to help better evaluate the diagnostic value of the variates.

However, there still exist some limitations: first, the study was retrospective in nature, and the indication bias for surgery could hardly be avoided; besides, the patient enrollment period was relatively short and therefore the sample size was comparatively not large enough. As a single-center study in a tertiary center, the results might not be generalized to all other dialysis patients, as patients in our center are mostly at the end-stage of the disease with severe symptoms and delayed surgical opportunity. Moreover, as always mentioned by other studies, ionized calcium and bone-specific ALP measurement were more accurate but also not available in our hospital. Finally, it is a pity that the examination result of serum 250HD was not included in the current study. This laboratory examination was not routinely performed in many hospitals in China, and most ESRD patients refuse this test as they were having heavy economic burden as expensive treatment with dialysis and relevant medication expand. We would consider including this examination in the future study.

In conclusion, this study for the first time, enrolled the most comprehensive relevant clinical parameters to screen the most valuable predictors for the SH following PTX. Currently, there exist a latency between the laboratory result of clinical symptoms or low serum calcium level and treatment. We hope this study would be helpful in identifying dialysis patients who are at greater risk of developing SH after PTX and thereby help clinicians in arriving at a fairly reliable forecast of post-operative hypocalcimia in the future, and thereby initiating the preventative treatment earlier. Besides, large sample size is warranted in verifying the current results.
